# The immune landscape of undifferentiated pleomorphic sarcoma

**DOI:** 10.3389/fonc.2022.1008484

**Published:** 2022-10-12

**Authors:** Rossana Lazcano, Carmelia M. Barreto, Ruth Salazar, Fernando Carapeto, Raymond S. Traweek, Cheuk H. Leung, Swati Gite, Jay Mehta, Davis R. Ingram, Khalida M. Wani, Kim-Anh T. Vu, Edwin R. Parra, Wei Lu, Jianling Zhou, Russell G. Witt, Brandon Cope, Prapassorn Thirasastr, Heather Y. Lin, Christopher P. Scally, Anthony P. Conley, Ravin Ratan, J. Andrew Livingston, Alexandra M. Zarzour, Joseph Ludwig, Dejka Araujo, Vinod Ravi, Shreyaskumar Patel, Robert Benjamin, Jennifer Wargo, Ignacio I. Wistuba, Neeta Somaiah, Christina L. Roland, Emily Z. Keung, Luisa Solis, Wei-Lien Wang, Alexander J. Lazar, Elise F. Nassif

**Affiliations:** ^1^ Translational Molecular Pathology, The University of Texas MD Anderson Cancer Center, Houston, TX, United States; ^2^ Surgical Oncology, The University of Texas MD Anderson Cancer Center, Houston, TX, United States; ^3^ Biostatistics, The University of Texas MD Anderson Cancer Center, Houston, TX, United States; ^4^ Sarcoma Medical Oncology, The University of Texas MD Anderson Cancer Center, Houston, TX, United States; ^5^ Thoracic/Head and Neck Medical Oncology, The University of Texas MD Anderson Cancer Center, Houston, TX, United States; ^6^ Division of Pathology and Laboratory Medicine, The University of Texas, MD Anderson Cancer Center, Houston, TX, United States; ^7^ Genomic Medicine, The University of Texas MD Anderson Cancer Center, Houston, TX, United States

**Keywords:** undifferentiated pleomorphic sarcoma, immune checkpoint inhibitors, adenosine pathway, immune microenvironment, tertiary lymphoid structures

## Abstract

**Introduction:**

Undifferentiated pleomorphic sarcoma (UPS) can be associated with a relatively dense immune infiltration. Immune checkpoint inhibitors (anti-PD1, anti-PDL1, and anti-CTLA4) are effective in 20% of UPS patients. We characterize the immune microenvironment of UPS and its association with oncologic outcomes.

**Material and methods:**

Surgically resected UPS samples were stained by immunohistochemistry (IHC) for the following: tumor-associated immune cells (CD3, CD8, CD163, CD20), immune checkpoints (stimulatory: OX40, ICOS; inhibitory: PD-L1, LAG3, IDO1, PD1), and the adenosine pathway (CD73, CD39). Sections were reviewed for the presence of lymphoid aggregates (LA). Clinical data were retrospectively obtained for all samples. The Wilcoxon rank-sum and Kruskal-Wallis tests were used to compare distributions. Correlations between biomarkers were measured by Spearman correlation. Univariate and multivariate Cox models were used to identify biomarkers associated with overall survival (OS) and disease-free survival (DFS). Unsupervised clustering was performed, and Kaplan-Meier curves and log-rank tests used for comparison of OS and DFS between immune clusters.

**Results:**

Samples analyzed (n=105) included 46 primary tumors, 34 local recurrences, and 25 metastases. LA were found in 23% (n=10/43), 17% (n=4/24), and 30% (n=7/23) of primary, recurrent, and metastatic samples, respectively. In primary UPS, CD73 expression was significantly higher after preoperative radiation therapy (*p*=0.009). CD39 expression was significantly correlated with PD1 expression (primary: *p*=0.002, recurrent: *p*=0.004, metastatic: *p*=0.001), PD-L1 expression (primary: *p*=0.009), and CD3+ cell densities (primary: *p*=0.016, recurrent: *p*=0.043, metastatic: *p*=0.028). In recurrent tumors, there was a strong correlation between CD39 and CD73 (*p=*0.015), and both were also correlated with CD163+ cell densities (CD39 *p=*0.013; CD73 *p<*0.001). In multivariate analyses, higher densities of CD3+ and CD8+ cells (Cox Hazard Ratio [HR]=0.33; *p*=0.010) were independently associated with OS (CD3+, HR=0.19, *p*<0.001; CD8+, HR= 0.33, *p*=0.010) and DFS (CD3+, HR=0.34, *p*=0.018; CD8+, HR=0.34, *p*= 0.014). Unsupervised clustering of IHC values revealed three immunologically distinct clusters: immune high, intermediate, and low. In primary tumors, these clusters were significantly associated with OS (log-rank *p*<0.0001) and DFS (*p*<0.001).

**Conclusion:**

We identified three immunologically distinct clusters of UPS Associated with OS and DFS. Our data support further investigations of combination anti-PD-1/PD-L1 and adenosine pathway inhibitors in UPS.

## Introduction

Undifferentiated pleomorphic sarcoma (UPS) is a soft tissue sarcoma with pleomorphic spindle cell histology that lacks lineage-specific differentiation (1–3). UPS accounts for 10-20% of all soft tissue sarcomas (4–6) and the current standard-of-care treatment for localized disease is surgical resection with wide margins at an expert sarcoma centers (7–10). Neoadjuvant radiation therapy (RT) improves local control (11, 12), while chemotherapy improves survival in a subset of UPS patients (13). Following multimodal treatment, local and distant recurrences occur in 13 – 42% and 31 – 45% of patients, respectively (11, 12, 14), therefore, new treatment strategies are needed to improve disease control.

Immune checkpoint blockade (ICB) has demonstrated activity in UPS, with response rates ranging from 20 to 40% in prospective phase II trials in the advanced setting (15–18). These strategies have been extended to earlier disease stage settings in a recent phase II trial of neoadjuvant ICB with concurrent RT (n=10), which demonstrated a 90% pathologic response rate (19, 20). Correlative studies from these trials demonstrated that the immune microenvironment was associated with response to ICB treatment and survival (21, 22). These translational analyses identified B-cells and tertiary lymphoid structures (TLS) as prognostic and predictive factors of response to ICB (22, 23), which has been confirmed in other cohorts and trials (24–26). TLS are organized lymphoid aggregates (LA) with a germinal center and similar structure to secondary lymphoid organs (27).

At the molecular level, UPS is characterized by a complex karyotype with extensive genomic rearrangements and numerous copy number alterations. The most frequent inactivating mutations are found in the tumor suppressor genes *TP53* (30%), *RB1* (21%), and *ATRX* (38%) (4). UPS, similar to other sarcomas with complex genomic profiles, can show extensive T-cell infiltration (15, 28, 29), oligoclonal T-cell repertoires, and increased expression of genes involved in antigen presentation and T-cell mediated apoptosis (29). The expression of PD-L1 reported in the literature is highly variable (17), ranging from 0 to almost three-quarters of cases, albeit generally displaying a low level of expression (13, 15). Overall, PD-L1 is inconsistently associated with response to ICB in sarcomas, and better biomarkers are needed to optimize patient selection for immunotherapies (21, 30).

Thus, a better understanding of the immune landscape of UPS may inform future ICB-based therapeutic strategies, stratification of patients, and design of biomarker-based novel clinical trials. The aims of this study were to describe the tumor immune microenvironment in a large cohort of UPS, to identify patterns of infiltration by tumor-associated immune cells (TAICs) and expression of immune checkpoints (IC) and their prognostic significance, and to build an immune-based classification system associated with oncologic outcomes.

## Methods

### Patient samples

We identified 105 formalin-fixed paraffin-embedded (FFPE) UPS surgical samples from patients treated at The University of Texas MD Anderson Cancer Center (MD Anderson; Houston, Texas, USA) between January 2004 and October 2010 whose tumor tissue samples had been previously formatted into tissue microarrays (TMA) (3). From this previously published cohort of 208 tumor samples, we excluded a TMA of exclusively radiation-induced UPS.

### Clinical characteristics

Clinical variables recorded included demographic characteristics such as age at diagnosis and sex. Disease-associated variables collected included tissue type (primary, locally recurrent or metastatic), tumor size, site of surgically resected disease (head and neck, trunk, lung, extremities), and histologic type of UPS (radiation-associated or not). Histologic grading was foregone due to the significant effects that perioperative therapies had on mitotic rate, percentage necrosis, and state of perceived differentiation. Perioperative treatment modalities were recorded, including preoperative chemotherapy (anthracycline-based or other regimens) and pre- and post-operative RT. For patients with multiple surgical resections, treatment modalities between each surgical resection were recorded (chemotherapy regimens and RT). The last known status of each patient was censored as of 04/15/22.

### Immunohistochemistry (IHC) staining

The TMA was generated from the FFPE UPS samples using two 1-mm-diameter cores as previously described (31). The UPS TMA was stained by IHC for the following biomarkers: TAICs (T-cells [CD3+], cytotoxic T-cells [CD8+], B-cells [CD20+], and macrophages [CD163+]), stimulatory IC (OX40, ICOS), inhibitory IC (PD-L1, LAG3, IDO1, PD1), and markers of the adenosine pathway (CD73, CD39). Immunohistochemical staining was performed on TMA tissue sections (4um) in a Leica Bond Max automated stainer (Leica Biosystems Nussloch GmbH). Briefly, tissue sections were deparaffinized and rehydrated following the Leica Bond protocol. Antigen retrieval was performed for 20 minutes with Bond Epitope Retrieval Solution #2 (Leica Biosystems, equivalent EDTA, pH 9.0) or Bond Epitope Retrieval Solution # 1 (Leica Biosystems, equivalent Citrate Buffer, pH 6.0). Primary antibodies were incubated for 15 minutes at room temperature and detected using the Bond Polymer Refine Detection kit (Leica Biosystems) with DAB as the chromogen. The slides were counterstained with hematoxylin, dehydrated, and cover-slipped. Antibody clones and their vendor information as well as dilution and antigen retrieval conditions, are summarized in **Supplementary Table 1**. All IHC slides were scanned using an Aperio AT2 (Leica Biosystems).

### Image analysis

The scanned UPS TMA slides stained with antibodies against CD3, CD8, CD20, CD163, PD1, ICOS, OX40, and IDO1 were analyzed using the HALO v3.1.1 (Indica Labs) digital image analysis software. Using TMA segmentation tools, each core was selected for analysis, excluding necrotic and artifact areas. Cell segmentation was performed based on the cytonuclear v2.0.5 algorithm for all the biomarkers except for OX40, for which the Area Quantification v2.2.1 algorithm was used. A threshold to detect positive immune cells was established by two pathologists (LS and RS), and the final analysis was reviewed to ensure that the software detected positive cells consistently. The results were expressed in cell densities (number of positive cells per area of analysis, cells/mm^2^) and area of analysis for OX40 (mm^2^). As there were two cores per sample, the mean density was calculated per sample. PD-L1 scoring was performed using direct microscope evaluation, and we used a cut-off of ≥ 1% of malignant cells with partial or complete membrane staining to classify tumors as positive and negative for this biomarker. For CD73 and CD39 IHC evaluation, we used direct microscope evaluation and recorded the percentage (0-100) of tumor membrane positivity.

In addition, we reviewed Hematoxylin-Eosin (H&E) whole-section slides to assess the presence of LA. This was done in concordance with previous work defining a minimum number of lymphoid cells necessary for LA (32). Additionally, previous work from our group has demonstrated no significant reduction in lymphoid infiltrate as a result of previous radiation therapy; as such, a group of more than 50 lymphoid cells located in the tumor area was used to define the presence of LA (33).This marker is not a surrogate for TLS, as no IHC markers of TLS were used.

### Statistical analysis

Clinical characteristics and biomarkers were summarized. The Chi-squared test or Fisher’s exact test was used to assess the association between two categorical variables. The Wilcoxon rank-sum test was used to compare the distributions between two groups. The Kruskal-Wallis test was used to determine if one group had different distributions from the others. Dwass-Steel-Critchlow-Fligner (DSCF) test provided the multiple comparisons between groups. Spearman correlation was used to evaluate the correlation between two continuous variables. Linear mixed effect model was used to find the association between continuous biomarkers and tissue type (primary, recurrent, metastatic). Box Cox transformation was applied if necessary. Generalized linear mixed effect model with logit function was used to find the association between binary biomarkers and tissue type (primary, recurrent, metastatic).

Unsupervised Ward-linkage hierarchical clustering based on Pearson correlation coefficient was performed in R using the Bioconductor’s Complex Heatmap package (34). The correlation matrices of single markers were generated in R using the corrplot mixed function from the Corrplot package.

Overall survival (OS) was calculated from the date of surgical resection to the date of death or last follow-up. Disease-free survival (DFS) was calculated from the date of surgical resection to the date of relapse, death, or last follow-up, whichever occurred first. The distributions of OS and DFS were estimated by the Kaplan-Meier method (35). Regression analyses of survival data based on the Cox proportional hazard (PH) regression model (36) were conducted on OS and PFS. Log-rank test (37) was performed to test the difference in survival between immune clusters. A two-sided p-value < 0.05 was considered statistically significant. SAS version 9.4 was used to carry out all analyses.

### Ethical approvals

This study was approved by the MD Anderson Institutional Review Board (LAB04-0890) and was conducted according to the principles of the Helsinki Declaration. A waiver of Informed Consent was approved because this is a retrospective project that involves no diagnostic or therapeutic intervention and no direct patient contact.

## Results

### Population description

Of the 105 samples identified, 46 were primary tumors, 34 were locally recurrent tumors resected from 25 patients, and 25 were metastatic tumors accounting for 24 patients. Ten patients had longitudinal surgical specimens collected. The median age at diagnosis was 65 years (interquartile range [IQR]: 53-74). In primary, recurrent, and metastatic UPS, the median tumor size was 6 cm (IQR: 3.5 cm – 10 cm), 4.35 cm (IQR: 3 cm – 6.9 cm), and 7.85 cm (IQR: 6.3 cm- 12 cm), respectively. The most common primary and recurrent locations were the lower extremities (primary n = 25, 54%; recurrent n=16, 47%) followed by the trunk (primary n = 13, 28%; recurrent n=13, 38%). The most common metastatic site was the lungs (n = 14, 56%). Neoadjuvant treatment for primary tumors consisted of chemotherapy for 9 patients (20%), RT for 14 patients (30%, median dose=50 Gy), and a combination of chemo-radiation for 5 patients (10.9%). Preoperative treatment for recurrent tumors consisted of chemotherapy in 14 cases (41%), RT in 15 cases (44%, median dose = 50 Gy), and a combination of both in 5 cases (14.7%). Preoperative treatment for metastatic lesions consisted of chemotherapy in 13 cases (52%), RT in 9 cases (36%, median dose=50 Gy), and a combination of both in 5 cases (20%). No patient received immune checkpoint blockade in this cohort. Additional clinicopathologic information is summarized in **Table 1**.

**Table 1:** Clinical characteristics of the cohort (primary, recurrence and metastasis).

Variable	Category	Primaryn = 46 (43.8%)	Recurrence*n = 25 (32.4%)	Metastasis** n = 24 (23.8%)
**Age at diagnosis (median, IQR)**		61.1 (53-73.75)	66.7 (65-82)	62.9 (53-77)
**Sex**	**Male**	20 (43)	13 (52)	13 (54)
	**Female**	26 (57)	12 (48)	11 (46)
**Histotype**	**UPS/MFH**	42 (91)	31 (91)	25 (100)
	**Radiation associated UPS**	4 (9)	3 (9)	0(0)
**Tumor size**	**< 5cm**	18 (39)	22 (65)	4 (16)
	**5 - 10cm**	19 (41)	8 (24)	10 (40)
	**> 10cm**	9 (20)	4 (12)	11 (44)
**Tumor site**	**Head/neck**	3 (7)	0(0)	0(0)
	**Trunk**	13 (28)	13 (38)	8 (32)
	**Lung**	0(0)	0(0)	14 (56)
	**Upper extremities**	5 (11)	5 (15)	2 (8)
	**Lower extremities**	25 (54)	16 (47)	1 (4)
**Preoperative chemotherapy**	**No**	37 (80)	20 (59)	12 (48)
	**Yes**	9 (20)	14 (41)	13 (52)
**Preoperative adriamycin-based chemotherapy**	**No**	4 (44)	6 (43)	9 (69)
	**Yes**	5 (56)	8 (57)	4 (31)
**Preoperative RT**	**No**	32 (70)	19 (56)	16 (64)
** **	**Yes**	14 (30)	15 (44)	9 (36)
**Postoperative RT**	**No**	31 (67)	23 (68)	13 (52)
** **	**Yes**	15 (33)	11 (32)	12 (48)

*5 patients had more than one recurrence sample, 25 patients accounted for 34 samples.

**1 patient had more than one metastasis sample, 24 patients accounted for 25 samples.

IQR, interquartile range; MFH, malignant fibrous histiocytoma; UPS, undifferentiated pleomorphic sarcoma.

### Association of immune biomarkers with clinical characteristics

**Figure 1:**
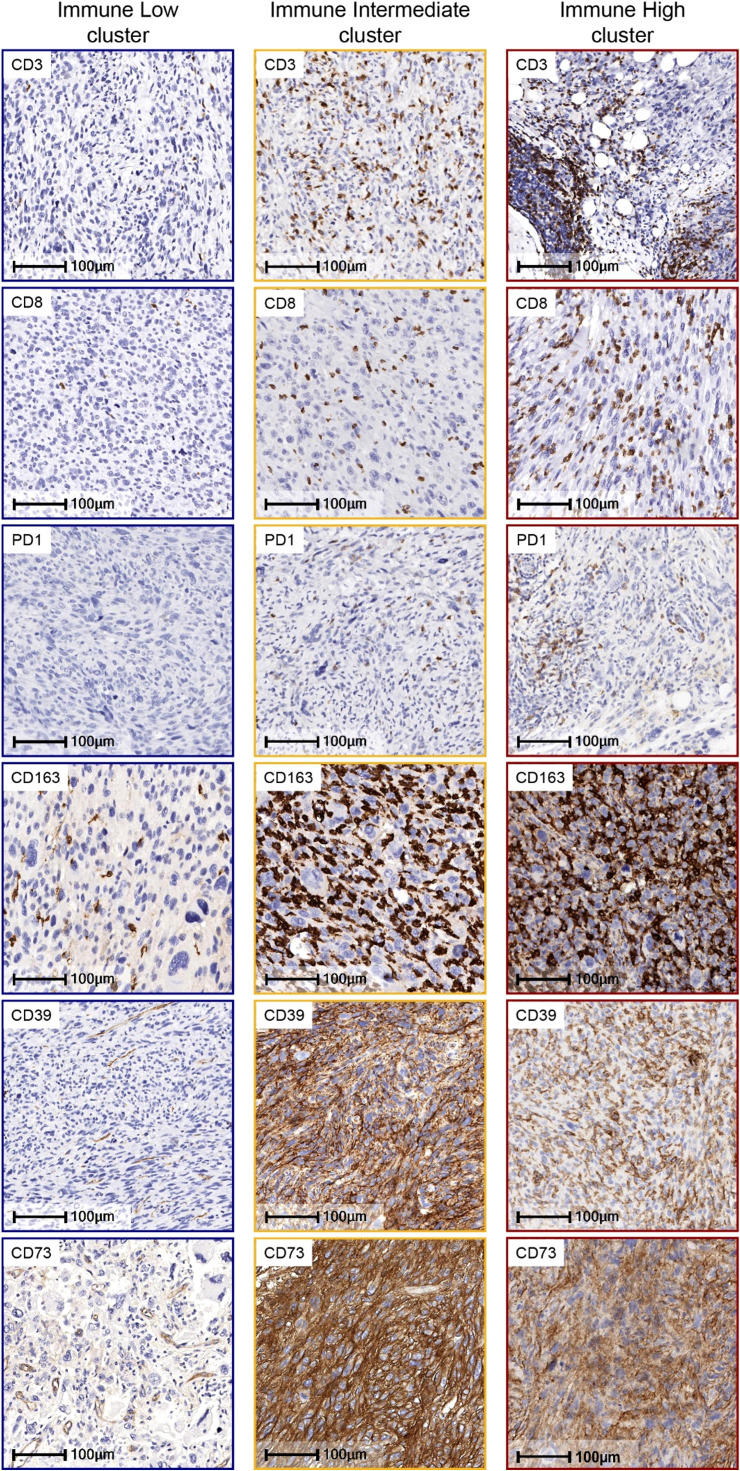
Representative Immunohistochemistry (IHC) Images of Tumor-Associated Immune Cells and Immune Checkpoints in UPS by Immune Clusters. On the left in blue square brackets, IHC images of UPS tumors expressing low cell density of CD3, CD8, PD1, CD163, and tumor cells negative for CD39 and CD73, which are characteristic of the Immune Low cluster samples. On the middle column in yellow square brackets IHC images of UPS tumors with moderate density of CD3, CD8 and PD1, and high density of macrophages expressing CD163, and tumor cells positive for CD39 and CD73, which is characteristic of the Immune Intermediate cluster. On the right column in red square brackets, IHC images of UPS tumors with high cell density of all the biomarkers shown, which are representative of the Immune High cluster, *IHC images are at 400x magnification*.

We first evaluated the presence and prevalence of TAICs, IC, and adenosine pathway biomarkers (**Figure 1**). Overall, CD163+ cells were the most abundant TAICs in UPS (median density: 2640 cells/mm^2^; IQR: 1728-3501 cells/mm^2^), followed by CD3+ (median density: 285 cells/mm^2^; IQR: 82-615 cells/mm^2^) and CD8+ cells (median density: 131 cells/mm^2^; IQR:42-360 cells/mm^2^). B-cells were less abundant (median density: 0.5 cells/mm^2^; IQR: 0-3 cells/mm^2^). LA were observed in 21 of the 90 samples evaluated (18.4%).

We next sought to evaluate the association of immune biomarkers with clinical features. TAICs densities and biomarker expression were not significantly different in primary, recurrent, and metastatic UPS (**Table 2**). In primary tumors, size was inversely correlated with ICOS and CD39 expression (ICOS r=-0.38, *p*=0.012; CD39 r=-0.33, *p*=0.024). In recurrent tumors, size was inversely correlated with CD3+ cell density (r=-0.48, *p*=0.011). In metastatic UPS, tumor size was inversely correlated with OX40 (r=-0.55, *p*=0.006). In metastatic tumors, age was significantly correlated with OX40 (r=0.46, *p*=0.025) and inversely correlated with IDO1 (r=-0.41, *p*=0.045; **Supplementary Table 2**). We did not find any significant differences with other clinical characteristics studied, including sex, age at diagnosis, and tumor site.

**Table 2:** TILs and immune checkpoint biomarker expression in UPS.

	Overall N = 105		Primary n = 46		Recurrence n = 34		Metastasis n = 25		
Biomarker	n	Median (IQR)	n	Median (IQR)	n	Median (IQR)	n	Median (IQR)	p-value*
**Tumor infiltrating lymphocytes (cells/mm^2^)**									
**CD3**	105	285 (82-615)	46	283 (77-460)	34	296 (82-551)	25	280 (112-862)	0.379
**CD8**	104	131 (42-360)	46	120 (40-234)	33	126 (43-452)	25	158 (53-565)	0.220
**CD20**	99	0 (0-3)	44	0.6 (0-2)	31	0 (0-2)	24	1.5 (0-4)	0.379
**CD163**	103	2640 (1728-3501)	46	2666 (1689-3467)	33	2861 (1929-3667)	24	4215.6 (1415-2951)	0.429
**Immune-checkpoint biomarkers:**									
**Stimulatory (cells/mm^2^)**									
**ICOS**	99	2 (0-13)	43	3.5 (0-12)	32	2.5 (0-18)	24	3.4 (0-12)	0.629
**OX40**	101	0 (0-2)	44	0 (0-1)	32	0.3	25	0 (0-2)	0.674
**Inhibitory (cells/mm^2^)**									
**LAG3**	102	0 (0-0)	44	0(0-0)	33	0 (0-18)	25	0 (0-0)	NA
**IDO1**	103	0 (0-0)	45	0 (0-0)	33	0 (0-0)	25	0 (0-0)	0.462
**PD1**	104	36 (8-125)	46	34.5 (6-107)	33	37 (11-81)	25	34.5 (13-158)	0.401
**Biomarkers expression by malignant cells(%)**									
**PD-L1**	104	0 (0-0)	45	0 (0-0)	34	0 (0-0)	25	0 (0-0)	0.462
**CD73**	102	56 (1-85)	44	58 (1-80)	33	80 (10-86)	25	15 (1-85)	0.276
**CD39**	105	5 (0.5-25)	46	2 (0-15)	34	8 (0.5-39)	25	10.5 (1-23)	0.402

*Kruskal-Wallis tests.

IQR, interquartile range; NA, Not applicable; TILs, tumor infiltrating lymphocytes; UPS, undifferentiated pleomorphic sarcoma.

Additionally, we investigated whether preoperative treatments were associated with immune features in the primary, recurrent, and metastatic settings. We observed significantly higher CD73 expression in primary tumors that received preoperative RT compared to those that did not receive preoperative RT (*p*=0.009; **Supplementary Table 3**). Preoperative RT and chemotherapy were not associated with any significant differences in immune biomarkers in the recurrent and metastatic setting (**Supplementary Tables 4, 5**).

### Correlations between biomarkers

**Figure 2:**
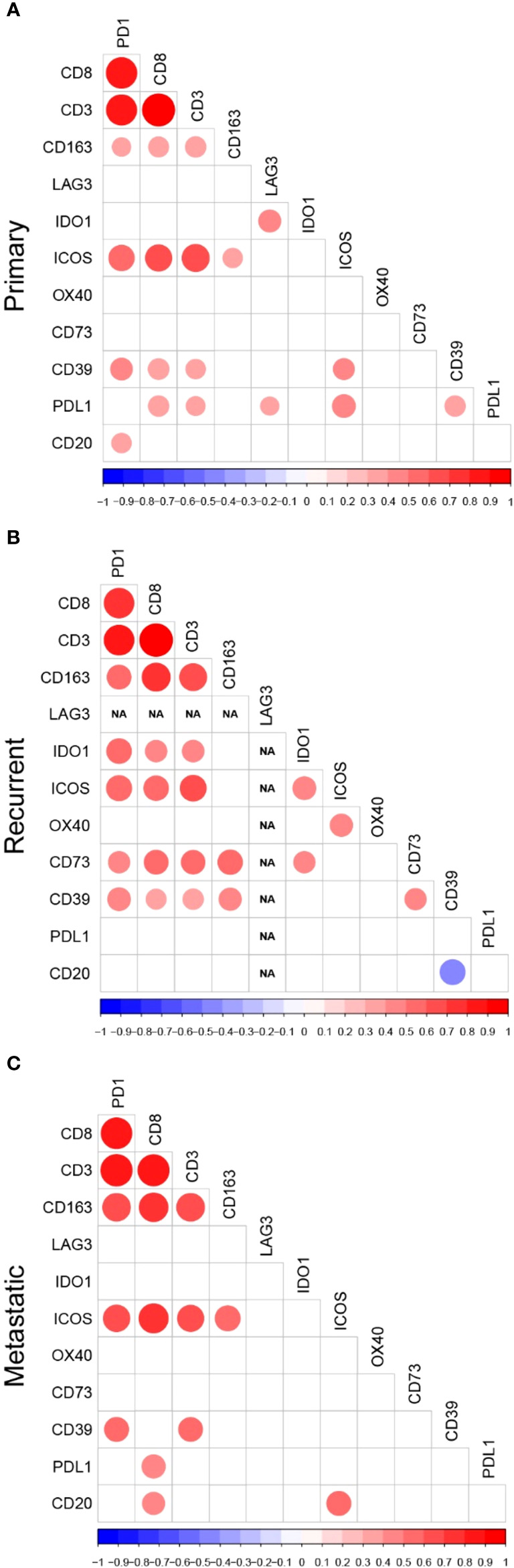
Correlation Plots of Immune Biomarkers Studied in **(A)** Primary, **(B)** Recurrent, and **(C)** Metastatic UPS. Correlation plots displaying only the statistically significant correlations between tumor-associated immune cells, immune checkpoints and adenosine pathway biomarkers in **(A)** primary, **(B)** recurrent, and **(C)** metastatic samples. *The colors of the circles are representative of the Spearman correlation with the scale represented at the bottom of the plots. The size of the circles indicate the significance level of the correlation, with larger circles representing lower p-values*.

Next, we investigated the correlations between these biomarkers in primary, recurrent, and metastatic settings, separately. Detailed significant correlations in each disease setting are represented in **Figure 2** and select correlations of interest are detailed in the following paragraphs.

In all three settings, CD3+, CD8+, and CD163+ cell densities and PD-1 expression were all significantly correlated with each other and with multiple other biomarkers in the primary, recurrent, and metastatic settings. However, the correlations between TAICs and other immune biomarkers varied according to setting. In primary tumors, CD3+ and CD8+ densities were significantly correlated with PD-L1 (CD3+ r=0.33 *p*=0.026; CD8+ r=0.39, *p*=0.009), CD39 (CD3+ r=0.35, *p*=0.016; CD8+ r=0.40, *p*=0.006), and ICOS (CD3+ r=0.67, *p*<0.001; CD8+ r=0.63, *p*=<0.001). In recurrent tumors, CD3+ and CD8+ densities, as well as PD-1 expression significantly correlated with most of the biomarkers except OX40, PD-L1, and B-cell densities, whereas CD163+ cell densities correlated only with the adenosine pathway biomarkers.

B-cell densities were correlated with very few immune biomarkers and these correlations were dependent on the disease setting. In primary tumors, B-cell densities were significantly correlated with PD1 (r=0.35, *p*=0.020). In recurrent UPS, B-cell densities were inversely correlated with CD39 (r=-0.46, *p*=0.027). In the metastatic setting, B-cell densities were significantly correlated with CD8+ cell density (r=0.43, *p*=0.039) and ICOS expression (r=0.50, *p*=0.018).

Among stimulatory IC, OX40 was correlated only with ICOS and only in the recurrent setting (r=0.65, *p<*0.001). In contrast, ICOS was significantly correlated with many immune biomarkers: CD3+ and CD8+ densities and PD1 in all settings, CD163+ densities in primary (r=0.35, *p=*0.022) and metastatic (r=0.53, *p=*0.011) tumors, CD39 (r=0.41, *p=*0.007) and PD-L1 (r= 0.48, *p=*0.001) expression in primary tumors, IDO1 expression (r=0.51, *p=*0.012) in recurrent tumors, and B-cell density (r=0.50, *p=*0.018) in metastatic setting.

Among the inhibitory IC, LAG3 was rarely expressed and thus, correlated with only one other inhibitory IC and only in the primary setting: IDO1 (r=0.45, *p=*0.002). IDO1 also had only few correlations with other biomarkers, except in the recurrent setting.

Among the biomarkers in the adenosine pathway, CD39 expression was significantly correlated with PD1 expression and CD3+ densities in all three settings. The correlations with other immune biomarkers were dependent on the disease setting. In primary UPS, CD39 expression also correlated with PD-L1 expression (r=0.39, *p*=0.009). In recurrent tumors, there was a strong correlation between both adenosine pathway biomarkers (CD39 and CD73 r=0.48, *p=*0.015), and both also correlated with CD163+ cell densities (CD39 r=0.49, *p=*0.013; CD73 *r=*0.64, *p<*0.001).

Additionally, we analyzed the correlation between biomarkers in primary tumors that received neoadjuvant chemotherapy or radiation therapy and found that the neoadjuvant treatment modified the interactions between biomarkers and the correlations were lost on the cases that received neoadjuvant treatment for many of the biomarkers **(** **Supplementary Table 6** **)**.

### Association of lymphoid aggregates with other immune biomarkers

Since TLS are important predictive biomarkers for response to ICB, we compared the immune infiltrate of samples with and without LA. In primary, recurrent, and metastatic samples, LA were present in 23% (n=10/43), 17% (n=4/24), and 30% (n=7/23), respectively. Primary tumors with LA had higher IDO1 expression compared than those without LA (*p*=0.019). Recurrent UPS with LA had higher densities of CD3+ (*p*=0.011) and CD163+ cells (*p*=0.029) and higher expressions of ICOS (*p*<0.001) and OX40 (*p*=0.020) compared to cases with no LA. Metastatic cases with LA had higher CD3+ cell densities compared to cases without LA (*p*=0.033**;** **Table 3**).

**Table 3:** Immune biomarkers associated with lymphoid aggregates.

Type of sample	Immune biomarkers (cells/mm^2^)	With LA		Without LA		
		n	Median (min, max)	n	Median (min, max)	p-value*
**Primary**	**IDO1**	10	(0,10.13)	32	0 (0,0.53)	**0.019**
**Recurrent**	**CD3**	4	884 (457, 2190)	20	270 (12.7, 1530)	**0.011**
	**ICOS**	4	56.8 (32.4, 78.1)	19	1.17 (0, 63.1)	**<.001**
	**OX40**	4	2.23 (0, 4.01)	19	0 (0, 3.24)	**0.020**
	**CD163**	4	3743 (3237, 4081)	20	2735 (689, 4348)	**0.029**
**Metastatic**	**CD3**	7	1006 (112, 1697)	16	250 (27.8, 2145)	**0.033**

*Wilcoxon rank-sum exact test. Bold values indicate significant p-value.

We next examined the correlation between immune biomarkers in the presence of LA. In primary tumors with LA, LAG3 was directly correlated with PD-L1 expression (r=1, *p*<0.001) and the only 2 cases with LAG-3 positive tumor cells had LA. In recurrent tumors with LA, OX40 expression was inversely correlated with CD3+ cell densities (r=-1, *p*<0.001). In metastatic cases with LA, PD-L1 expression was directly correlated to CD39 expression (r=0.76, *p*=0.049) and IDO1 (r=0.76, *p*=0.046), while in metastatic tumors without LA, we did not find any correlation between PD-L1 or any other biomarker studied. Correlations between biomarkers in samples without LA followed different patterns (**Supplementary Figure 1**).

### Association of the immune infiltrate in primary tumors with prognosis

Next, we investigated the prognostic impact of immune biomarkers in primary tumors only, since recurrent and metastatic tumors were more heterogeneous with respect to disease course and multimodal management. First, we identified clinical variables significantly associated with OS and DFS in multivariate analyses, which were histologic type (radiation-associated or not) and size of the primary tumor (**Tables 4**, **5**). Then, we ran univariate analyses for each biomarker separately. Subsequently, we selected the biomarkers associated with survival in univariate analyses and included them in a multivariate Cox model adjusted for histotype and tumor size.

**Table 4:** Univariate and multivariate cox analyses for overall survival in primary tumors.

Parameter	Category	Death	Total	Univariate HR (95% CI)	p-value	Multivariate HR (95% CI)	p-value
**Diagnosis age (years)**		27	46	1.03 (1.00, 1.05)	**0.049**		
**Sex**	**Male**	13	20	Ref			
	**Female**	14	26	1.18 (0.55, 2.52)	0.675		
**Histotype**	**UPS/MFH**	23	42	Ref		Ref	
	**Radiation associated UPS**	4	4	3.24 (1.06, 9.87)	**0.039**	3.91 (1.23, 12.5)	**0.021**
**Site**	**Lower extremities**	13	25	Ref			
	**Others**	14	21	1.65 (0.77, 3.55)	0.200		
**Size**	**< 5cm**	7	18	Ref		Ref	
	**5 - 10cm**	12	19	2.66 (1.04, 6.81)	**0.042**	2.50 (0.97, 6.42)	0.058
	**> 10cm**	8	9	4.41 (1.58, 12.3)	**0.004**	5.03 (1.78, 14.2)	**0.002**
**Preoperative chemotherapy**	**No**	22	37	Ref			
	**Yes**	5	9	0.98 (0.37, 2.61)	0.966		
**Preoperative RT**	**No**	20	32	Ref			
	**Yes**	7	14	0.64 (0.27, 1.51)	0.306		
**LA**	**No**	20	33	Ref			
	**Yes**	4	10	0.55 (0.19, 1.60)	0.272		
Biomarkers*							
**CD3**	**Low**	18	23	Ref		Ref	
	**High**	9	23	0.20 (0.09, 0.47)	**<.001**	0.19 (0.07, 0.50)	**<.001**
**CD8**	**Low**	18	23	Ref		Ref	
	**High**	9	23	0.26 (0.11, 0.59)	**0.001**	0.33 (0.14, 0.76)	**0.010**
**CD20**	**Low**	12	22	Ref			
	**High**	14	22	1.29 (0.59, 2.79)	0.522		
**ICOS**	**Low**	15	21	Ref		Ref	
	**High**	10	22	0.39 (0.17, 0.88)	**0.024**	0.62 (0.24, 1.60)	0.322
**OX40**	**Low**	17	28	Ref			
	**High**	9	16	1.01 (0.45, 2.28)	0.977		
**LAG3**	**Low**	26	43	Ref			
	**High**	0	1	NA	NA		
**IDO1**	**Low**	24	41	Ref			
	**High**	2	4	0.83 (0.20, 3.51)	0.798		
**PD1**	**Low**	16	23	Ref			
	**High**	11	23	0.45 (0.21, 0.99)	**0.046**	0.34 (0.14, 0.83)	**0.018**
**PDL1**	**Low**	23	36	Ref			
	**High**	4	9	0.46 (0.16, 1.35)	0.157		
**CD73**	**Low**	15	22	Ref			
	**High**	11	22	0.82 (0.38, 1.79)	0.618		
**CD39**	**Low**	15	23	Ref			
	**High**	12	23	0.70 (0.33, 1.51)	0.364		
**CD163**	**Low**	17	23	Ref		Ref	
	**High**	10	23	0.35 (0.15, 0.78)	**0.010**	0.54 (0.23, 1.31)	0.174

*Each biomarker was categorized as high or low based on the median and if significantly associated with survival in univariate analysis was subsequently included in a backward model adjusted for histotype and tumor size.

95%CI, 95% confidence interval; HR, hazard ratio; LA, lymphoid aggregates; MFH, malignant fibrous histiocytoma; RT, radiation therapy; UPS, undifferentiated pleomorphic sarcoma. Bold values indicate significant p-value.

**Table 5:** Univariate and multivariate cox analyses for disease-free survival in primary tumors.

Parameter	Category	Event	Total	Univariate HR (95% CI)	p-value	Multivariate HR (95% CI)	p-value
**Diagnosis age (years)**		27	46	1.02 (1.00, 1.05)	0.060		
**Sex**	**Male**	13	20	Ref			
	**Female**	14	26	0.87 (0.41, 1.86)	0.725		
**Histotype**	**UPS/MFH**	23	42	Ref		Ref	
	**Radiation associated UPS**	4	4	3.59 (1.17, 11.0)	**0.025**	4.62 (1.43, 14.9)	**0.011**
**Site**	**Lower extremities**	13	25	Ref			
	**Others**	14	21	1.30 (0.61, 2.76)	0.500		
**Depth**	**superficial**	12	18	Ref			
	**deep**	15	28	0.74 (0.35, 1.59)	0.445		
**Size**	**< 5cm**	7	18	Ref		Ref	
	**5 - 10cm**	12	19	2.63 (1.02, 6.77)	**0.045**	2.41 (0.93, 6.27)	0.070
	**> 10cm**	8	9	5.91 (2.10, 16.6)	**<0.001**	6.86 (2.41, 19.5)	**<0.001**
**Preoperative chemotherapy**	**No**	22	37	Ref			
	**Yes**	5	9	1.00 (0.38, 2.65)	0.999		
**Preoperative RT**	**No**	20	32	Ref			
	**Yes**	7	14	0.72 (0.30, 1.71)	0.459		
**LA**	**No**	20	33	Ref			
	**Yes**	4	10	0.46 (0.16, 1.36)	0.161		
Biomarkers*							
**CD3**	**Low**	18	23	Ref		Ref	
	**High**	9	23	0.29 (0.13, 0.67)	**0.003**	0.34 (0.14, 0.83)	**0.018**
**CD8**	**Low**	18	23	Ref		Ref	
	**High**	9	23	0.30 (0.13, 0.69)	**0.005**	0.34 (0.14, 0.81)	**0.014**
**CD20**	**Low**	12	22	Ref			
	**High**	14	22	1.40 (0.64, 3.03)	0.398		
**ICOS**	**Low**	15	21	Ref			
	**High**	10	22	0.50 (0.22, 1.14)	0.100		
**OX40**	**Low**	17	28	Ref			
	**High**	9	16	1.04 (0.46, 2.35)	0.917		
**LAG3**	**Low**	26	43	Ref			
	**High**	0	1	NA	NA		
**IDO1**	**Low**	24	41	Ref			
	**High**	2	4	0.64 (0.15, 2.71)	0.545		
**PD1**	**Low**	16	23	Ref			
	**High**	11	23	0.59 (0.27, 1.28)	0.185		
**PDL1**	**Low**	23	36	Ref			
	**High**	4	9	0.61 (0.21, 1.78)	0.362		
**CD73**	**Low**	15	22	Ref			
	**High**	11	22	0.78 (0.36, 1.71)	0.539		
**CD39**	**Low**	15	23	Ref			
	**High**	12	23	0.61 (0.28, 1.31)	0.201		
**CD163**	**Low**	17	23	Ref			
	**High**	10	23	0.46 (0.21, 1.02)	0.055		

*Each biomarker was categorized as high or low based on the median and if significantly associated with survival in univariate analysis was subsequently included in a backward model adjusted for histotype and tumor size.

95%CI, 95% confidence interval; HR, hazard ratio; LA, lymphoid aggregates; MFH, malignant fibrous histiocytoma; NA, Not Applicable; RT, radiation therapy; UPS, undifferentiated pleomorphic sarcoma. Bold values indicate significant p-value.

In univariate analyses, higher densities of CD3+ (Hazard Ratio [HR]=0.20, *p*<0.001), CD8+ (HR=0.26, *p*=0.001), and CD163+ cells (HR= 0.35, *p*=0.010) and higher expression of ICOS (HR=0.39, *p*=0.024) and PD1 (HR=0.45; *p*=0.046) were significantly associated with better OS. In multivariate analyses, higher densities of CD3+ (HR=0.19, *p*<0.001) and CD8+ cells (HR=0.33, *p*=0.010) remained independently associated with OS (**Table 4**). Regarding DFS, in univariate analyses, only densities of CD3+ (HR=0.29, *p*=0.003) and CD8+ cells (HR=0.30, *p*=0.005) were associated with improved DFS and both remained independently associated with DFS in multivariate analysis (CD3+ HR=0.34, *p*=0.018; CD8+ HR=0.34, *p*=0.014; **Table 5**).

### Unsupervised cluster analysis identifies three distinctive and prognostic immune clusters

We sought to build an immune-based classification of UPS tumors. To this end, the expression levels of IC biomarkers and the abundance of TAICs were analyzed with unsupervised hierarchical clustering in primary, recurrent, and metastatic UPS, separately. LAG3, PD-L1, and IDO1 were excluded from the clustering algorithm because their median expression was 0 in all three disease settings.

**Figure 3:**
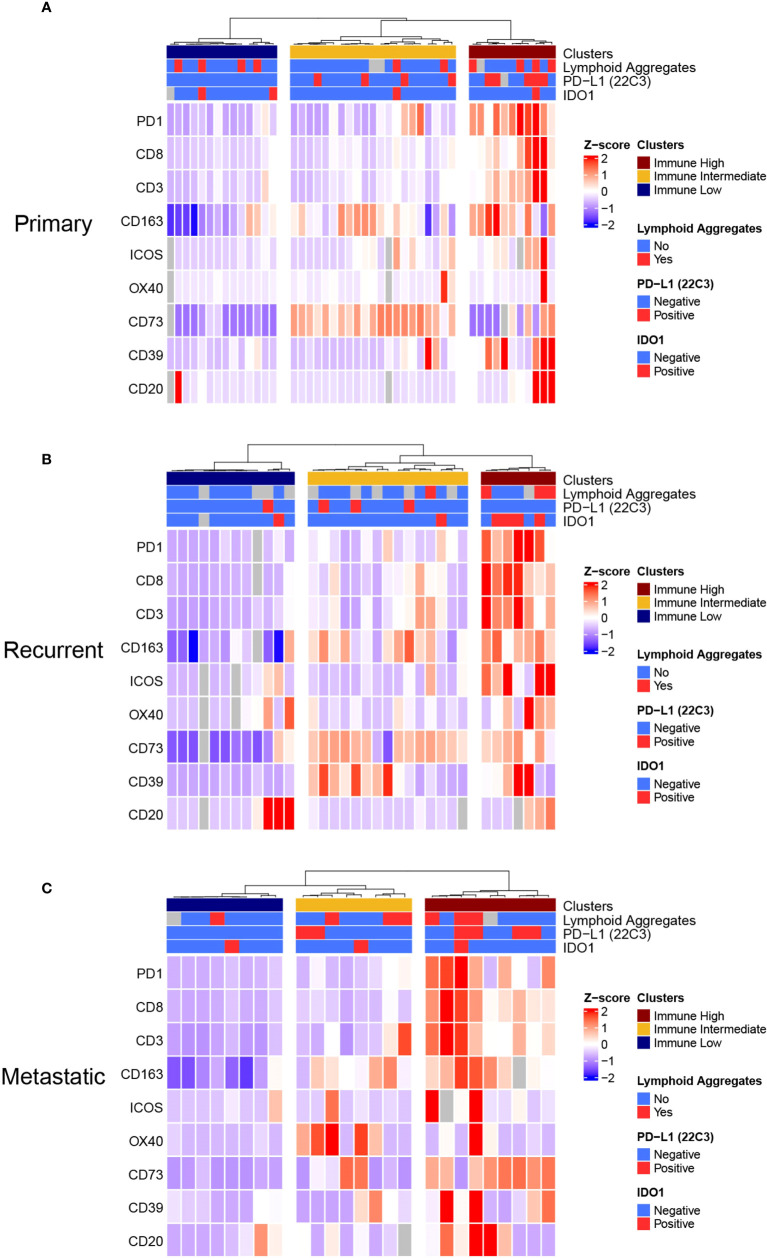
Unsupervised Hierarchical Clustering Heatmaps of **(A)** Primary, **(B)** Recurrent, and **(C)** Metastatic UPS displays Three Distinctive Immune Clusters. **(A)**, Primary, **(B)** recurrent and **(C)** metastatic UPS samples were clustered in three distinctive clusters: Immune Low, Immune Intermediate, and Immune High clusters. *This analysis was done by unsupervised Ward-linkage hierarchical clustering based on Pearson correlation coefficient*.

In all three settings (primary, recurrent, and metastatic), three clusters were obtained that described differential expression of immune biomarkers: immune low, immune intermediate, and immune high. These clusters seemed to follow similar patterns in primary, recurrent, and metastatic samples, although the intermediate cluster differed slightly in the metastatic setting (**Figure 3**). For most immune biomarkers studied (CD3, CD8, PD1, ICOS), expression was significantly lower in immune low clusters and highest in immune high clusters in primary, recurrent, and metastatic samples. B-cells had significantly higher densities in the immune high cluster only in the primary setting (Kruskal-Wallis *p*=0.012). CD163+ cell densities were significantly lower in the immune low clusters (Kruskal-Wallis primary: *p*=0.002; recurrent: *p*<0.001, metastatic: *p*=0.002), but not significantly lower in the immune intermediate clusters compared to the immune high clusters (DSCF primary: *p*=0.24; recurrent: *p*=0.33, metastatic: *p*=0.31). OX40 was not differentially expressed between clusters neither in primary, recurrent, nor metastatic settings. The adenosine pathway biomarkers (CD73 and CD39) did not follow the same distributions as the other immune parameters. CD73 was significantly higher in the immune intermediate cluster followed by the immune high cluster in the primary and recurrent settings (Kruskal-Wallis primary: *p*<0.001; recurrent: *p*=0.19). In recurrent UPS, CD39 was also highest in the immune intermediate cluster followed by the immune high cluster (Kruskal-Wallis *p*=0.019; **Supplementary Table 7**).

We investigated whether these clusters were modified by disease trajectories with longitudinal biospecimens that were available for ten patients. Overall, we observed that the immune clusters of UPS samples changed over time and with treatments. We did not identify any specific pattern in these cluster changes which could be attributed to time or treatment modalities (**Supplementary Figure 2**).

We then sought to investigate associations between these clusters and clinical characteristics and preoperative treatments. In the primary setting, neoadjuvant RT was significantly associated with immune intermediate cluster (RT: immune low n=0/14[0%], immune intermediate n=11/14 [79%], immune high: n=3/14 [21%], *p*=0.002). Immune clusters were not associated with any other characteristics studied (sex, age, tumor size, tumor site, preoperative chemotherapy) neither in primary, recurrent, nor metastatic settings. **(** **Supplementary Table 8** **).**

**Figure 4:**
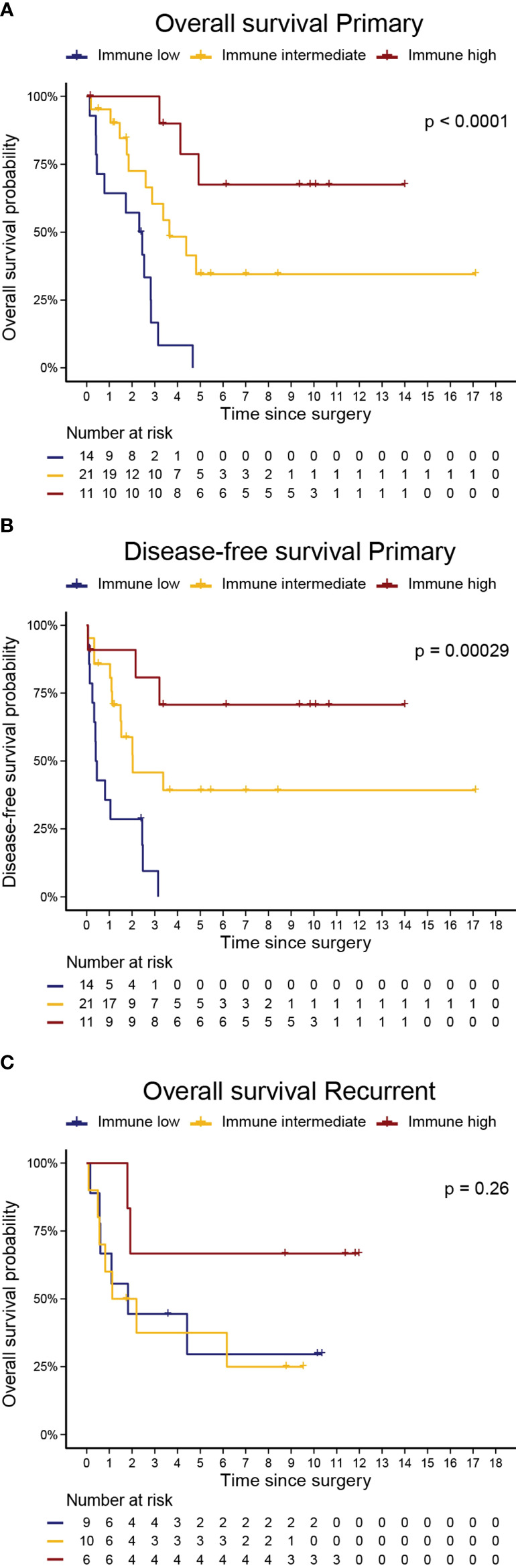
Immune Clusters are associated with Overall Survival and Disease-Free Survival in Primary UPS. Kaplan Meier curves of **(A)** overall survival and **(B)** disease-free survival in primary UPS, and **(C)** overall survival in recurrent UPS. *P-values are log-rank test for comparison of survival curves*.

In primary UPS, the immune clusters were significantly associated with both OS (log-rank *p*<0.0001) and DFS (*p*<0.001). The median OS was 29 months, 44.3 months, and not reached in the immune low, intermediate, and high clusters, respectively. The median DFS was 5.2 months, 24.7 months, and not reached in the immune low, intermediate, and high clusters, respectively. In the recurrent setting, there was a trend for better OS in the immune high cluster (log-rank *p*=0.26; **Figure 4**).

To investigate these trends in survival more thoroughly, we performed a Kaplan-Meier survival analysis examining OS and PFS by immune cluster among primary tumors that received neoadjuvant therapies and those that did not. Among primary tumors, the immune high cluster demonstrated significantly improved OS when compared to immune intermediate and immune low clusters in both samples that received neoadjuvant therapies (log-rank, *p* = 0.00063) and in samples that did not (log-rank, *p* = 0.0016). Similarly, the immune high cluster demonstrated significantly improved PFS over the other immune clusters in both samples that received neoadjuvant therapies (log-rank, *p* = 0.0092) and in samples that did not (log-rank, *p* = 0.0051; **Supplementary Figure 3**).

## Discussion

This study is one of the largest to describe the immune landscape of UPS tumor tissue samples, including densities of TAICs and the expression levels of IC biomarkers and adenosine pathway in primary, recurrent, and metastatic UPS. Further, we investigated the association of these biomarkers with survival and obtained an immune-based classification of UPS tumors, with a significant prognostic impact in primary UPS.

Our study shows improved survival in primary UPS with higher tumor infiltration by T-cells and cytotoxic T-cells. This observation is consistent with previous reports that used different methodologies to assess the presence of T-cells and cytotoxic T-cells (38–41). In contrast to other studies, PD-L1 was not associated with survival in primary UPS in this cohort. The prognostic impact of PD-L1 in sarcoma is controversial and variable (42–45). These discrepancies may be explained by methodological differences but also by the fact that the tumor microenvironment hosts complex interactions between immune cells, IC, and tumor cells. Thus, the prognostic impact of the tumor immune infiltration is not driven by a single immune variable.

To address this, we developed an immune classification system integrating multiple immune biomarkers. Our three-way cluster-based classification was significantly associated with both OS and DFS in primary tumors. The predictive value for response and survival with ICB of this classification will be investigated in future studies. Previously, our group demonstrated that soft-tissue sarcomas, including UPS, could be subtyped in 5 sarcoma immune classes (SIC), labeled SIC A, B, C, D, and E, using transcriptomic data, and that these SIC were predictive of response and survival with ICB in the advanced setting (23). The tumor immune microenvironment differed significantly by SIC: SIC A being immune low, SIC C being dominated by a vascular signature, SIC E being immune high, and SICs B and D being more heterogeneous, with generally immune low and immune high profiles, respectively. Overall, the expression of genes associated with T-cells was highest in SICs D and E, intermediate in SICs B and C, and very low in SIC A. The expression of IC genes was highest in SICs E and D, and low to very low in the other SICs. We will compare the SIC genomic predictive classification and our three cluster IHC-based prognostic classification in future work. Additionally, we will investigate spatial distribution and intratumor heterogeneity of the immune microenvironment and immune classes using novel technologies (46).

In the SIC classification, the strongest predictive factor for response and survival with ICB was the presence of TLS and B-cells in the tumor microenvironment (23). Thus, we evaluated the presence of LA and B-cells in our present report. Neither B-cell density nor LA was significantly associated with survival. However, B-cell densities were overall low, the number of samples with LA was small, and none of the patients in our cohort were treated with ICB. Furthermore, B-cells are known to be particularly radiosensitive (47) and more than a third of our surgical samples were collected after preoperative RT. Additionally, LA were not evaluated with markers specific to TLS, but with H&E slides displaying LA of more than 50 lymphocytes, which is a limitation of our evaluation. The field of TLS in cancer is evolving and we are now moving towards more precise assessments of TLS and B-cells (27). Characterization of TLS markers will be included in future work.

We described specific correlations of immune therapeutic targets in the tumor microenvironment of UPS, in each particular disease setting, as well as in the context of LA. Some of our observations, if confirmed, may provide a rationale for clinical trials testing novel combinations of ICB in UPS. For instance, in primary tumors, LAG3 was correlated with PDL1 in the presence of LA, and LAG3 was only expressed in samples with LA. These observations are consistent with prior reports (11) and the addition of anti-LAG3 therapies to anti-PD1/PDL1 treatment may be more effective in primary UPS with TLS (48). Likewise, novel immunotherapeutic drugs targeting the adenosine pathway are being developed. CD39 expression was significantly correlated with PD1 expression and T-cell densities in all three settings and CD73 had a higher expression after neoadjuvant RT. These observations may suggest a synergistic potential of combining drugs targeting the adenosine pathway with anti-PDL1/PD1 drugs (49, 50), particularly in combination with RT (51, 52). The immune intermediate cluster had higher adenosine pathway expression and may therefore have the greatest benefit from this approach.

Our report is retrospective, and interpretations are limited by its descriptive nature. Notably, the differences observed in immune microenvironment after specific treatments or between primary, recurrent, and metastatic tumors are exploratory by nature and would warrant longitudinal paired analyses to properly assess the role of treatments (33, 53) and time (54, 55). The heterogeneity of our cohort impacts our results most notably regarding preoperative treatments received and we have tried to address this by detailing associations in specific subgroups; however, subgroup analyses are limited by the smaller number of patients. We have not evaluated other immune biomarkers of interest, including vascular markers, which were significantly associated with SIC C and may be higher in our immune intermediate cluster (23), T-regulatory cells for which data regarding their prognostic and predictive impact is discordant in the literature (21, 22, 40, 56), macrophage phenotypes (57, 58), and other IC biomarkers such as TIM3 and TIGIT.

In conclusion, this report provides a detailed description of the immune landscape of UPS and its association with oncologic outcomes. Our data confirm the prognostic impact of the immune microenvironment of UPS and may further help in identifying a rationale combination of immunotherapeutic drugs to be tested and selection of patients who are more likely to benefit from these combinations.

## Data availability statement

The authors declare that the data supporting the findings of this study are available within the manuscript and its supplementary information files. All other relevant de-identified data related to the current study are available from the corresponding author (EFN) upon reasonable academic request and will require the researcher to sign a data access agreement with the University of Texas MD Anderson Cancer Center after approval.

## Ethics statement

This study was approved by the MD Anderson Institutional Review Board (LAB04-0890) and was conducted according to the principles of the Helsinki Declaration. A waiver of Informed Consent was approved because this is a retrospective project that involves no diagnostic or therapeutic intervention and no direct patient contact.

## Author contributions

RL: Formal Analysis, Investigation, Methodology, Validation, Visualization, Writing - Original Draft Preparation, Writing – Review and Editing. CB: Formal Analysis, Investigation, Methodology, Validation, Writing - Original Draft Preparation. RS: Methodology. FC: Formal Analysis, Investigation, Methodology, Validation, Visualization, Writing - Original Draft Preparation. RT: Data Curation, Visualization. CL: Formal Analysis, Methodology. SG: Methodology. JM: Writing – Review and Editing. DI: Resources. KW: Resources. K-AV: Visualization. EP: Validation. WL: Methodology-Validation. JZ: Methodology. RW: Writing – Review and Editing. BC: Writing – Review and Editing. PT: Writing – Review and Editing. HL: Formal Analysis, Methodology. AC: Writing – Review and Editing. RR: Writing – Review and Editing. AL: Writing – Review and Editing. AZ: Writing – Review and Editing. DA: Writing – Review and Editing. SP: Writing – Review and Editing. RB: Writing – Review and Editing. VR: Writing – Review and Editing. CS: Writing – Review and Editing. JW: Writing – Review and Editing. IW: Writing – Review and Editing. NS: Writing – Review and Editing. CR: Writing – Review and Editing. EK: Writing – Review and Editing. LS: Writing – Review and Editing. W-LW: Conceptualization, Funding Acquisition, Investigation, Methodology, Resources, Supervision, Visualization, Writing – Review and Editing. AL: Conceptualization, Funding Acquisition, Investigation, Methodology, Project Administration, Resources, Supervision, Visualization, Writing – Original Draft Preparation, Writing – Review and Editing. EN: Data Curation, Investigation, Methodology, Supervision, Visualization, Writing – Original Draft Preparation, Writing – Review and Editing. All authors contributed to the article and approved the submitted version.

## Funding

EN declares research support from Fondation pour la Recherche Medicale and Fondation Nuovo-Soldati. RT declares research support from the National Institute of Health (T32CA009599). This project was supported in part by the Translational Molecular Pathology-Immuno-profiling laboratory Moonshot Platform (TMP-IL) at the Department Translational Molecular Pathology at the University of Texas MD Anderson Cancer.

## Acknowledgments

The authors thank the members of the Translational Molecular Pathology Immune-Profiling Laboratory (TMP-IL) for their able assistance.

## Conflict of interest

The authors declare that the research was conducted in the absence of any commercial or financial relationships that could be construed as a potential conflict of interest.

## Publisher’s note

All claims expressed in this article are solely those of the authors and do not necessarily represent those of their affiliated organizations, or those of the publisher, the editors and the reviewers. Any product that may be evaluated in this article, or claim that may be made by its manufacturer, is not guaranteed or endorsed by the publisher.
