# Supplementary material for: The immune landscape of undifferentiated pleomorphic sarcoma
Source: Front Oncol. 2022 Oct 12;12:1008484. doi: 10.3389/fonc.2022.1008484 (PMC9597628; doi:10.3389/fonc.2022.1008484)
Supplement: Supplementary file 1 [file DataSheet_1.docx]

Supplementary Material

# Supplementary Figures and Tables

## Supplementary Tables

**Supplementary Table 1. Immunohistochemistry clones used and conditions**

| **Marker** | **Manufacturer** | **Catalog number** | **Clone** | **Dilution** | **Control tissue** | **Antigen retrieval** |
| --- | --- | --- | --- | --- | --- | --- |
| **CD3** | Dako | A0452 |  | 1:100 | tonsil | ER1 (citrate), 20 min |
| **CD8** | Life Technology | MS457s |  | 1:100 | tonsil | ER2 (Tris-EDTA), 20 min |
| **CD20** | Dako | M075501-2 | L26 | 1:1400 | tonsil | ER1 (citrate), 20 min |
| **CD163** | Leica | NCL-L-CD163 | 10D6 | 1:100 | tonsil | ER1 (citrate), 20 min |
| **ICOS** | Cell signaling | 89601 | D1K2T | 1:100 | tonsil | ER1 (citrate), 20 min |
| **IDO1** | Cell signaling | 86630 | D5J4E | 1:2000 | tonsil | ER2 (Tris-EDTA), 5 min |
| **LAG3** | Cell signaling | 15372 | D2G40 | 1:100 | tonsil | ER1 (citrate), 20 min |
| **OX40** | eBioscience | 14-1347 | ACT-35 | 1:100 | tonsil | ER1 (citrate), 20 min |
| **PD1** | Abcam | ab137132 |  | 1:250 | tonsil | ER1 (citrate), 20 min |
| **PDL1** | Dako | M365329-2 | 22C3 | 1:50 | tonsil | ER2 (Tris-EDTA), 20 min |
| **CD39** | Abcam | ab223843 | EPR204 | 1:500 | tonsil | ER1 (citrate), 20 min |
| **CD73** | Cell Signaling | 13160S | D7F9A | 1:200 | tonsil | ER2 (Tris-EDTA), 20 min |

ER, Epitope retrieval solution

**Supplementary Table 2. Immune biomarkers associated with clinical characteristics**

| **Clinicopathological Data** | **Immune infiltrates (cell/mm2)** | **Spearman Correlation Coefficient** | **p-value** |
| --- | --- | --- | --- |
| **Size (cm)** | | | |
| Primary | ICOS | -0.381 | **0.012** |
|  | CD39 | -0.332 | **0.024** |
| Recurrent | CD3 | -0.476 | **0.011** |
| Metastatic | OX40 (mm^2^) | -0.547 | **0.006** |
| **Age** | | | |
| Metastatic | OX40 (mm^2^) | 0.457 | **0.025** |
|  | IDO1 | -0.413 | **0.045** |

## Supplementary Table 3. Comparison of Biomarker Expression across Neoadjuvant Treatments in Primary Tumors.

| **Biomarker** | **Preop Radiation Therapy** | | | | **p-value*** | **Preop Chemotherapy** | | | | **p-value*** |
| --- | --- | --- | --- | --- | --- | --- | --- | --- | --- | --- |
|  | **Yes** | | **No** | |  | **Yes** | | **No** | |  |
|  | **n** | **median (min, max)** | **n** | **median (min, max)** |  | **n** | **median (min, max)** | **n** | **median (min, max)** |  |
| **CD3** | 14 | 252 (45.3, 1079) | 32 | 290.6 (3.49, 3467.1) | 0.915 | 9 | 105 (48.7, 942) | 37 | 303 (3.49, 3467) | 0.478 |
| **CD8** | 14 | 127 (11.9, 934) | 32 | 114.6 (0.59, 2207) | 0.953 | 9 | 96.9 (16.8, 261) | 37 | 120 (0.59, 2207) | 0.586 |
| **CD20** | 14 | 0.26 (0, 3.6) | 30 | 0.60 (0, 71.4) | 0.129 | 9 | 0.53 (0, 8.44) | 35 | 0.57 (0, 71.4) | 0.831 |
| **ICOS** | 14 | 10.5 (0, 28.7) | 29 | 1.16 (0, 138.2) | 0.091 | 9 | 1.32 (0, 16.6) | 34 | 3.65 (0, 138) | 0.653 |
| **OX40** | 14 | 0.26 (0, 28.4) | 30 | 0 (0, 85.4) | 0.259 | 9 | 0 (0, 28.4) | 35 | 0 (0, 85.4) | 0.971 |
| **LAG3** | 14 | 0 (0, 0) | 30 | 0 (0, 0.52) | 1.00 | 9 | 0 (0, 0) | 35 | 0 (0, 0.52) | 1.00 |
| **IDO1** | 14 | 0 (0, 0) | 31 | 0 (0, 2.13) | 0.294 | 9 | 0 (0, 0) | 36 | 0 (0, 2.13) | 0.569 |
| **PD1** | 14 | 51.7 (4.26, 192) | 32 | 25.0 (0, 319.6) | 0.362 | 9 | 27.2 (3.62, 171) | 37 | 41.8 (0, 320) | 0.913 |
| **PDL1** | 13 | 0 (0, 25) | 32 | 0 (0, 80.0) | 0.400 | 9 | 0 (0, 25.0) | 36 | 0 (0, 80.0) | 0.868 |
| **CD73** | 13 | 80.0 (0, 96.5) | 31 | 10.5 (0, 95.0) | **0.009** | 9 | 55.0 (0, 96.5) | 35 | 60.0 (0, 95.0) | 0.891 |
| **CD39** | 14 | 2.0 (0, 98.0) | 32 | 2.5 (0, 85.0) | 0.724 | 9 | 8.0 (0, 35.0) | 37 | 1.0 (0, 98.0) | 0.643 |
| **CD163** | 14 | 3073 (507, 4986) | 32 | 2342 (246, 4703) | 0.113 | 9 | 2683 (1311, 3954) | 37 | 2650 (246, 4986) | 0.935 |

*Wilcoxon test

## Supplementary Table 4. Comparison of Biomarker Expression across Neoadjuvant Treatments in Recurrent Tumors.

| **Biomarker** | **Preop Radiation Therapy** | | | | **p-value*** | **Preop Chemotherapy** | | | | | **p-value*** |
| --- | --- | --- | --- | --- | --- | --- | --- | --- | --- | --- | --- |
|  | **Yes** | | **No** | |  | **Yes** | | | **No** | |  |
|  | **n** | **median (min, max)** | **n** | **median (min, max)** |  | **n** | **median (min, max)** | **n** | | **median (min, max)** |  |
| **CD3** | 12 | 504 (35.6, 2190) | 13 | 255 (12.7, 1530) | 0.470 | 9 | 550 (12.7, 2190) | 16 | | 190 (35.6, 1050) | 0.169 |
| **CD8** | 12 | 365 (4.56, 1768) | 13 | 56.8 (8.1, 1077) | 0.470 | 9 | 371 (8.1, 1768) | 16 | | 53.7 (4.56, 1069) | 0.084 |
| **CD20** | 12 | 0 (0, 16.2) | 11 | 0 (0, 30.4) | 0.658 | 7 | 0 (0, 1.12) | 16 | | 0 (0, 30.4) | 0.33 |
| **ICOS** | 12 | 1.36 (0, 78.1) | 12 | 6.62 (0, 29.8) | 0.841 | 9 | 2.32 (0, 45.9) | 15 | | 5.02 (0, 78.1) | 0.859 |
| **OX40** | 12 | 0 (0, 4.01) | 12 | 0 (0, 3.97) | 0.976 | 9 | 0 (0, 2.31) | 15 | | 0 (0, 4.01) | 0.537 |
| **LAG3** | 12 | 0 (0, 0) | 13 | 0 (0, 0) | 1.00 | 9 | 0 (0, 0) | 16 | | 0 (0, 0) | 1.00 |
| **IDO1** | 12 | 0 (0, 226.3) | 13 | 0 (0, 1.89) | 0.858 | 9 | 0 (0, 1.89) | 16 | | 0 (0, 226.3) | 0.603 |
| **PD1** | 12 | 58.8 (1.14, 232) | 13 | 29.7 (0.53, 400) | 0.470 | 9 | 125 (0.53, 400) | 16 | | 27.3 (1.14, 232.2) | 0.136 |
| **PDL1** | 12 | 0 (0, 0) | 13 | 0 (0, 20.0) | 0.480 | 9 | 0 (0, 0) | 16 | | 0 (0, 20.0) | 0.520 |
| **CD73** | 12 | 80.0 (0, 98.0) | 13 | 80.0 (0, 100) | 0.946 | 9 | 80.0 (0, 100) | 16 | | 65 (0, 98.0) | 0.813 |
| **CD39** | 12 | 5.25 (0, 75.0) | 13 | 0.5 (0, 85.0) | 0.776 | 9 | 25.0 (0, 85.0) | 16 | | 0.5 (0, 70.0) | 0.167 |
| **CD163** | 12 | 3189 (689, 4081) | 13 | 2637 (714, 4348) | 0.538 | 9 | 3143 (1360, 4348) | 16 | | 2599 (689, 4081) | 0.169 |

*Wilcoxon test

## Supplementary Table 5. Comparison of Biomarker Expression across Neoadjuvant Treatments in Metastatic Tumors.

| **Biomarker** | **Preop Radiation Therapy**  **No** | | | | **p-value*** | **Preop Chemotherapy**  **No** | | | | **p-value*** |
| --- | --- | --- | --- | --- | --- | --- | --- | --- | --- | --- |
|  | **Yes** | | | **No** |  | **Yes** | | **No** | |  |
|  | **n** | **median (min, max)** | **N** | **median (min, max)** |  | **N** | **median (min, max)** | **N** | **median (min, max)** |  |
| **CD3** | 9 | 280 (42.5, 1619) | 15 | 568 (27.8, 2145) | 1.00 | 12 | 254 (27.8, 1697) | 12 | 592 (29.2, 2145) | 0.551 |
| **CD8** | 9 | 128 (27.1, 591) | 15 | 213 (6.52, 2154) | 0.558 | 12 | 146 (6.52, 1308) | 12 | 259 (12.8, 2154) | 0.514 |
| **CD20** | 8 | 2.68 (0, 11.4) | 15 | 0 (0, 13.4) | 0.199 | 11 | 2.35 (0, 11.4) | 12 | 0.95 (0, 13.4) | 0.975 |
| **ICOS** | 9 | 4.85 (0, 9.33) | 14 | 4.39 (0, 106) | 0.475 | 12 | 1.92 (0, 102) | 11 | 5.29 (0, 106) | 0.345 |
| **OX40** | 9 | 0.62 (0, 5.91) | 15 | 0 (0, 7.97) | 0.427 | 12 | 0 (0, 5.91) | 12 | 0 (0, 7.97) | 0.998 |
| **LAG3** | 9 | 0 (0, 0) | 15 | 0 (0, 1.06) | 1.00 | 12 | 0 (0, 0) | 12 | 0 (0, 1.06) | 1.00 |
| **IDO1** | 9 | 0 (0, 2.77) | 15 | 0 (0, 0.61) | 0.355 | 12 | 0 (0, 2.77) | 12 | 0 (0, 0.62) | 0.739 |
| **PD1** | 9 | 34.6 (12.5, 290) | 15 | 43.0 (0.54, 706) | 1.00 | 12 | 27.0 (0.54, 706) | 12 | 54.7 (0.57, 455) | 0.630 |
| **PDL1** | 9 | 0 (0, 5.0) | 15 | 0 (0, 80.0) | 0.217 | 12 | 0 (0, 40.0) | 12 | 0 (0, 80.0) | 0.748 |
| **CD73** | 9 | 7.5 (0, 95.0) | 15 | 32.5 (0, 99.0) | 0.401 | 12 | 20.0 (0, 99.0) | 12 | 20.0 (0, 98.0) | 0.943 |
| **CD39** | 9 | 10.5 (0, 50.0) | 15 | 10.0 (0, 85.0) | 0.825 | 12 | 9.25 (0, 55.0) | 12 | 13.8 (0, 85.0) | 0.466 |
| **CD163** | 9 | 2116 (790, 3593) | 14 | 2401 (424, 4216) | 1.00 | 12 | 2157 (424, 4216) | 11 | 2640 (790, 4043) | 0.347 |

*Wilcoxon test

**Suppplementary table 6. Correlations between biomarkers in samples that received Neoadjuvant chemotherapy and/or radiation therapy.**

**A.Spearman correlation coefficient**

| **Biomarkers** | **CD3** | **CD8** | **CD20** | **ICOS** | **OX40** | **LAG3** | **IDO1** | **PD1** | **PDL1** | **CD73** | **CD39** | **CD163** |
| --- | --- | --- | --- | --- | --- | --- | --- | --- | --- | --- | --- | --- |
| **CD3** | 1 |  |  |  |  |  |  |  |  |  |  |  |
| **CD8** | 0.938 | 1 |  |  |  |  |  |  |  |  |  |  |
| **CD20** | 0.083 | -0.014 | 1 |  |  |  |  |  |  |  |  |  |
| **ICOS** | 0.477 | 0.420 | 0.185 | 1 |  |  |  |  |  |  |  |  |
| **OX40** | -0.266 | -0.389 | -0.084 | 0.304 | 1 |  |  |  |  |  |  |  |
| **LAG3** | NA | NA | NA | NA | NA | NA |  |  |  |  |  |  |
| **IDO1** | NA | NA | NA | NA | NA | NA | NA |  |  |  |  |  |
| **PD1** | 0.880 | 0.909 | 0.084 | 0.526 | -0.393 | NA | NA | 1 |  |  |  |  |
| **PDL1** | 0.212 | 0.278 | 0.274 | 0.473 | 0.186 | NA | NA | 0.311 | 1 |  |  |  |
| **CD73** | 0.064 | 0.043 | -0.171 | 0.110 | 0.107 | NA | NA | 0.258 | -0.08 | 1 |  |  |
| **CD39** | -0.038 | 0.029 | 0.036 | 0.182 | -0.160 | NA | NA | 0.199 | 0.302 | -0.022 | 1 |  |
| **CD163** | 0.476 | 0.418 | 0.404 | 0.456 | 0.050 | NA | NA | 0.383 | 0.490 | -0.206 | -0.127 | 1 |

**B. p-values of the coefficient**

| **Biomarkers** | **CD3** | **CD8** | **CD20** | **ICOS** | **OX40** | **LAG3** | **IDO1** | **PD1** | **PDL1** | **CD73** | **CD39** | **CD163** |
| --- | --- | --- | --- | --- | --- | --- | --- | --- | --- | --- | --- | --- |
| **CD3** |  |  |  |  |  |  |  |  |  |  |  |  |
| **CD8** | **<.001** |  |  |  |  |  |  |  |  |  |  |  |
| **CD20** | 0.743 | 0.956 |  |  |  |  |  |  |  |  |  |  |
| **ICOS** | **0.045** | 0.083 | 0.463 |  |  |  |  |  |  |  |  |  |
| **OX40** | 0.285 | 0.111 | 0.742 | 0.221 |  |  |  |  |  |  |  |  |
| **LAG3** | NA | NA | NA | NA | NA |  |  |  |  |  |  |  |
| **IDO1** | NA | NA | NA | NA | NA | NA |  |  |  |  |  |  |
| **PD1** | **<.001** | **<.001** | 0.740 | **0.025** | 0.106 | NA | NA |  |  |  |  |  |
| **PDL1** | 0.415 | 0.280 | 0.287 | 0.055 | 0.475 | NA | NA | 0.224 |  |  |  |  |
| **CD73** | 0.807 | 0.869 | 0.511 | 0.675 | 0.684 | NA | NA | 0.318 | 0.761 |  |  |  |
| **CD39** | 0.882 | 0.908 | 0.887 | 0.470 | 0.525 | NA | NA | 0.427 | 0.239 | 0.934 |  |  |
| **CD163** | **0.046** | 0.084 | 0.096 | 0.057 | 0.842 | NA | NA | 0.117 | **0.046** | 0.428 | 0.614 |  |

**Supplementary Table 7. Comparison of Biomarker Expression across Clusters in Primary, Recurrent, and Metastatic Tumors**

|  | **Immune low** | | **Immune intermediate** | | **Immune high** | |  |
| --- | --- | --- | --- | --- | --- | --- | --- |
| **Biomarkers** | **n** | **median**  **(min, max)** | **n** | **median**  **(min, max)** | **n** | **median**  **(min, max)** | **p-value*** |
| **Primary** | | | | | | | |
| **CD3** | 14 | 102 (3.5, 730) | 21 | 210 (22.7, 473) | 11 | 883 (372, 3467) | **<0.001** |
| **CD8** | 14 | 46.3 (0.6, 239) | 21 | 111 (4, 304) | 11 | 362 (173, 2207) | **<0.001** |
| **CD20** | 13 | 1.1 (0, 55.2) | 20 | 0 (0, 1.7) | 11 | 5.2 (0, 71.4) | **0.012** |
| **ICOS** | 13 | 0 (0, 7.5) | 20 | 3.8 (0, 31.2) | 10 | 13.9 (1.2, 138.2) | **0.002** |
| **OX40** | 13 | 0 (0, 2.3) | 20 | 0.3 (0, 28.4) | 11 | 0 (0, 85.4) | 0.193 |
| **LAG3** | 13 | 0 (0, 0) | 20 | 0 (0, 0) | 11 | 0 (0, 0.5) | 0.223 |
| **IDO1** | 13 | 0 (0, 0.5) | 21 | 0 (0, 2.1) | 11 | 0 (0, 0.5) | 0.598 |
| **PD1** | 14 | 9.5 (0, 82) | 21 | 22.3 (1.2, 171) | 11 | 149 (66.3, 320) | **<0.001** |
| **PDL1** | 14 | 0 (0, 0) | 21 | 0 (0, 80) | 10 | 2.5 (0, 10) | **0.020** |
| **CD73** | 13 | 1 (0, 30.5) | 21 | 80 (40, 96.5) | 10 | 10 (0, 92.5) | **<0.001** |
| **CD39** | 14 | 0.5 (0, 21) | 21 | 1 (0, 98) | 11 | 35 (1, 85) | **0.001** |
| **CD163** | 14 | 1583 (246, 3511) | 21 | 2774 (507, 3954) | 11 | 3644 (1027, 4986) | **0.002** |
| **Recurrent** | | | | | | | |
| **CD3** | 9 | 78.5 (12.7, 255) | 10 | 411 (35.6, 933) | 6 | 1050 (457, 2190) | **<0.001** |
| **CD8** | 9 | 37.5 (4.6, 56.8) | 10 | 248 (30.6, 656) | 6 | 969 (452, 1768) | **<0.001** |
| **CD20** | 9 | 0 (0, 30.4) | 9 | 0 (0, 3.1) | 5 | 0 (0, 16.2) | 0.696 |
| **ICOS** | 8 | 0.3 (0, 28.9) | 10 | 1.7 (0, 32.4) | 6 | 54.5 (11.2, 78.1) | **0.004** |
| **OX40** | 8 | 0 (0, 4) | 10 | 0 (0, 2.3) | 6 | 2.3 (0, 4) | 0.117 |
| **LAG3** | 9 | 0 (0, 0) | 10 | 0 (0, 0) | 6 | 0 (0, 0) | NA |
| **IDO1** | 9 | 0 (0, 1.5) | 10 | 0 (0, 0.5) | 6 | 1.5 (0, 226.3) | **0.012** |
| **PD1** | 9 | 8.6 (0.5, 56.2) | 10 | 35.6 (4.2, 127) | 6 | 203 (80.6, 400) | **<0.001** |
| **PDL1** | 9 | 0 (0, 5) | 10 | 0 (0, 20) | 6 | 0 (0, 0) | 0.720 |
| **CD73** | 9 | 1.5 (0, 80) | 10 | 88.8 (0, 98) | 6 | 85 (50, 100) | **0.002** |
| **CD39** | 9 | 0.5 (0, 5.5) | 10 | 27.5 (0, 75) | 6 | 22.5 (0, 85) | **0.019** |
| **CD163** | 9 | 1360 (689, 2637) | 10 | 3189 (2354, 3950) | 6 | 3733 (2833, 4348) | **<0.001** |
| **Metastatic** | | | | | | | |
| **CD3** | 7 | 53.7 (29.2, 254) | 8 | 272 (27.8, 1619) | 9 | 1006 (615, 2145) | **<0.001** |
| **CD8** | 7 | 27.1 (6.5, 136) | 8 | 141 (16, 359) | 9 | 789 (453, 2154) | **<0.001** |
| **CD20** | 7 | 0.6 (0, 7.1) | 7 | 1.2 (0, 3.7) | 9 | 3.7 (0, 13.4) | 0.360 |
| **ICOS** | 7 | 0 (0, 36) | 8 | 1.9 (0, 58.5) | 8 | 13 (0.6, 105.6) | **0.044** |
| **OX40** | 7 | 0 (0, 0.6) | 8 | 3.5 (0, 6.5) | 9 | 0 (0, 8) | 0.088 |
| **LAG3** | 7 | 0 (0, 1.1) | 8 | 0 (0, 0) | 9 | 0 (0, 0) | 0.297 |
| **IDO1** | 7 | 0 (0, 0.6) | 8 | 0 (0, 2.8) | 9 | 0 (0, 0.6) | 0.966 |
| **PD1** | 7 | 7.1 (0.6, 43) | 8 | 50.4 (0.5, 158) | 9 | 297 (19.5, 706) | **0.004** |
| **PDL1** | 7 | 0 (0, 0) | 8 | 0 (0, 5) | 9 | 0 (0, 80) | 0.098 |
| **CD73** | 7 | 2 (0, 15) | 8 | 18.8 (0, 99) | 9 | 85 (1, 98) | **0.013** |
| **CD39** | 7 | 8 (0.5, 20) | 8 | 5.3 (0, 50) | 9 | 22.5 (0, 85) | 0.149 |
| **CD163** | 7 | 1101 (424, 2385) | 8 | 2467 (1579, 3593) | 8 | 2960 (2199, 4216) | **0.002** |

* Kruskal-Wallis tests

## Supplementary Table 8. Comparison of Clinicopathologic Characteristics across Clusters in Primary, Recurrent and Metastatic samples

| **Variable** | **Level** | **Immune low** | **Immune intermediate** | **Immune high** | **p-value** |
| --- | --- | --- | --- | --- | --- |
| **Sex** | Male | 5 (25) | 9 (45) | 6 (30) | 0.640 |
|  | Female | 9 (35) | 12 (46) | 5 (19) |  |
| **Tumor size** | <5cm | 3 (17) | 8 (44) | 7 (39) | 0.193 |
|  | 5-10cm | 7 (37) | 8 (42) | 4 (21) |  |
|  | >10cm | 4 (44) | 5 (56) | 0 (0) |  |
| **Tumor site** | Head/neck | 2 (67) | 1 (33) | 0 (0) | 0.445 |
|  | Trunk | 5 (38) | 6 (46) | 2 (15) |  |
|  | Upper extremities | 0 (0) | 4 (80) | 1 (20) |  |
|  | Lower extremities | 7 (28) | 10 (40) | 8 (32) |  |
| **Depth** | Superficial | 5 (28) | 9 (50) | 4 (22) | 0.928 |
|  | Deep | 9 (32) | 12 (43) | 7 (25) |  |
| **Neoadjuvant chemotherapy** | No | 11 (30) | 16 (43) | 10 (27) | 0.717 |
|  | Yes | 3 (33) | 5 (56) | 1 (11) |  |
| **Neoadjuvant radiation** | No | 14 (44) | 10 (31) | 8 (25) | **0.002** |
|  | Yes | 0 (0) | 11 (79) | 3 (21) |  |
| **Lymphoid aggregates** | No | 10 (30) | 17 (52) | 6 (18) | 0.192 |
|  | Yes | 4 (40) | 2 (20) | 4 (40) |  |

## Supplementary Figures


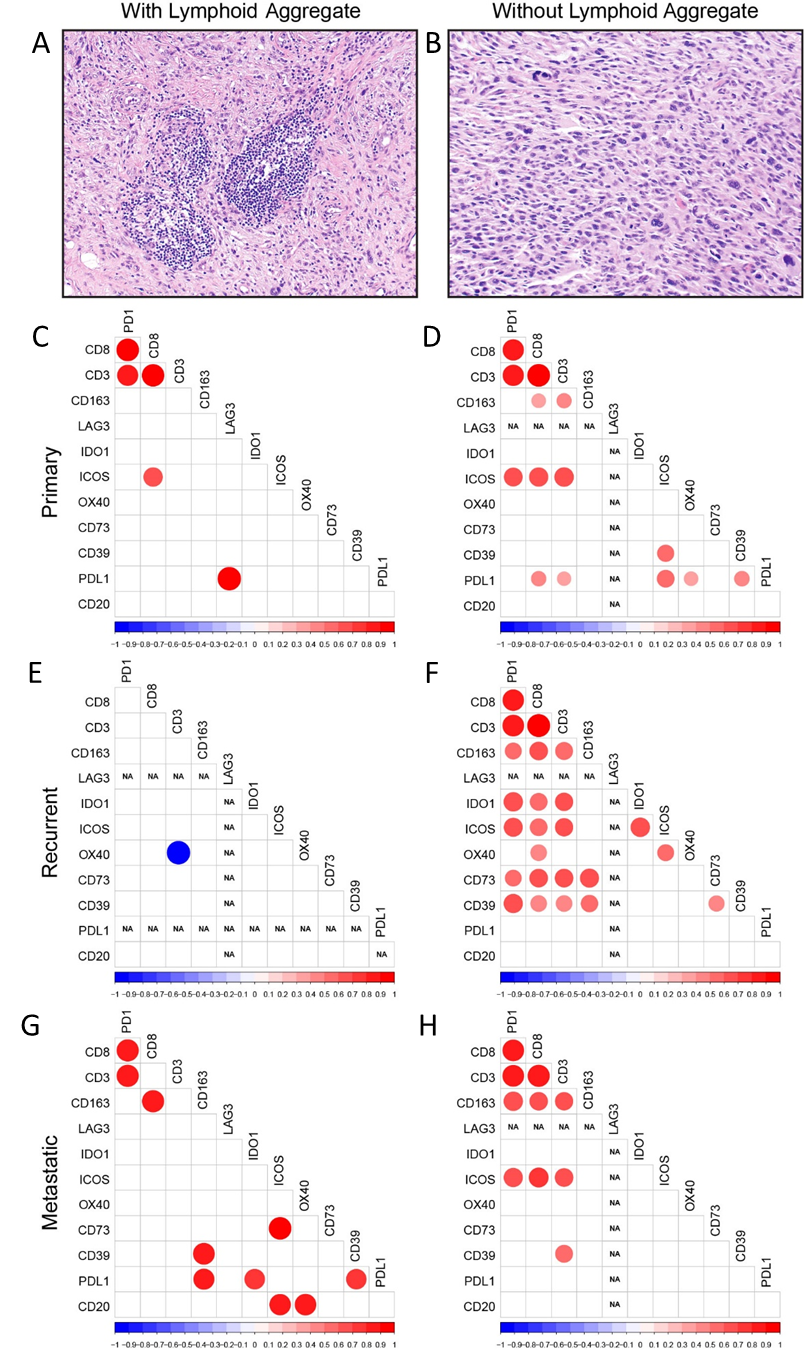


**Supplementary Figure 1. Correlation Plots with or without Lymphoid Aggregates (LA)**

H&E slide at 200x magnification showing an example of UPS tumor **(A)** with two LAs or **(B)** no LA.

Correlation plots in cases with LA in **(C)** primary, **(E)** recurrent**,** and **(G)** metastatic samples and in cases with no LA in **(D)** primary, **(F)** recurrent**,** and **(H)** metastatic samples. *The colors of the circles are representative of the Spearman correlation with the scale represented at the bottom of the plots. The size of the circles indicate the significance level of the correlation, with larger circles representing lower p-values.*


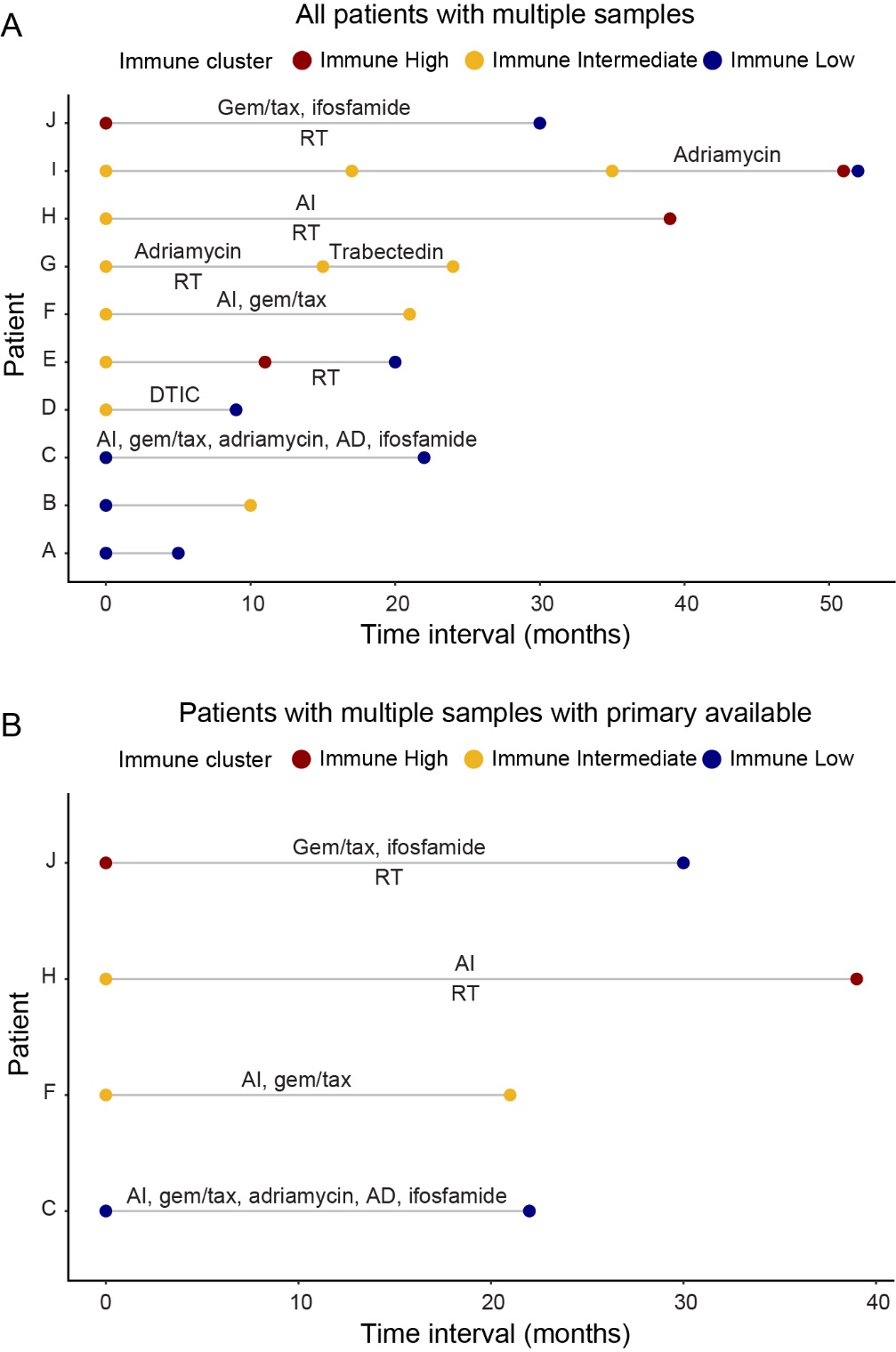
**Supplementary Figure 2. Longitudinal Patient-Level Representation of Changes in Immune Cluster over Time with Intervening Therapies**

**(A)** Overview of all patients with multiple samples available**. (B)** Overview of samples obtained from patients where primary tissue sampling was performed at MDACC**.** A total of 10 patients had multiple samples available. Dots represent timepoints at which a tumor sample was obtained. Color of the dot indicates immune cluster. Intervening systemic therapies are listed above each line connecting tumor sampling timepoints; radiation therapy is listed below each line.

*AI, doxorubicin and ifosfamide; gem/tax, gemcitabine and docetaxel; AD, doxorubicin and dacarbazine; RT, radiation therapy*

*
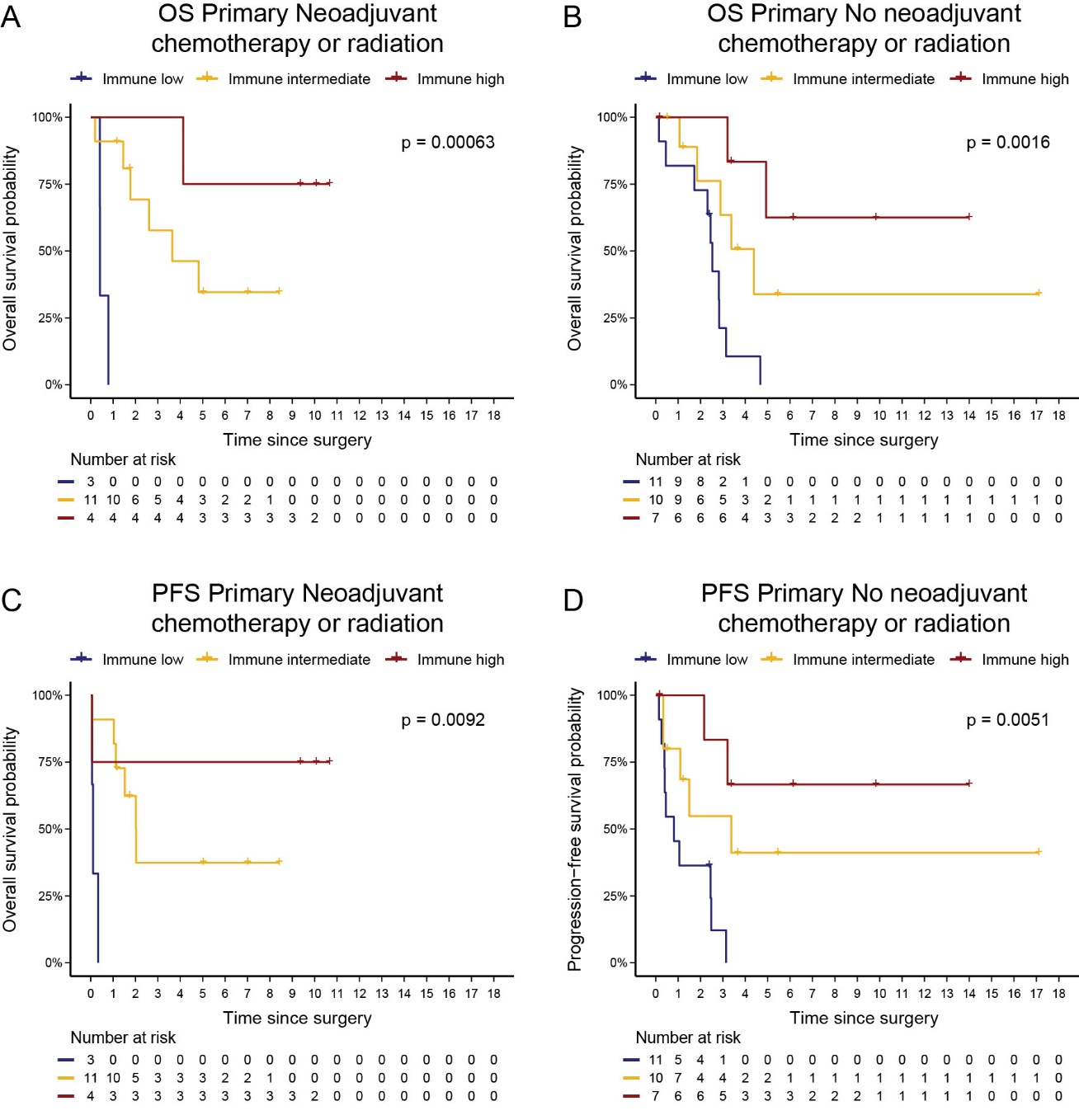
*

**Supplementary Figure 3.**

**Patients that received Neoadjuvant Chemotherapy and/or Radiotherapy show Immune Clusters are associated with Overall Survival and Disease-Free Survival in Primary UPS**

Kaplan Meier curves of **(A)** overall survival in patients who received neoadjuvant chemotherapy and/or radiation therapy and **(B)** patients who did not receive neoadjuvant therapy. **(C)** Disease-free survival in patients who received neoadjuvant chemotherapy and/or radiation therapy, and **(D)** patients who did not receive neoadjuvant therapy in primary UPS. *P-values are log-rank test for comparison of survival curves.*
